# Noise, Age, and Gender Effects on Speech Intelligibility and Sentence Comprehension for 11- to 13-Year-Old Children in Real Classrooms

**DOI:** 10.3389/fpsyg.2019.02166

**Published:** 2019-09-25

**Authors:** Nicola Prodi, Chiara Visentin, Erika Borella, Irene C. Mammarella, Alberto Di Domenico

**Affiliations:** ^1^Department of Engineering, University of Ferrara, Ferrara, Italy; ^2^Department of General Psychology, University of Padova, Padua, Italy; ^3^Department of Developmental and Social Psychology, University of Padova, Padua, Italy; ^4^Department of Psychological, Health and Territorial Sciences, University of Chieti, Chieti, Italy

**Keywords:** classroom acoustics, intelligibility, sentence comprehension, listening effort, noise, children, gender, response times

## Abstract

The present study aimed to investigate the effects of type of noise, age, and gender on children’s speech intelligibility (SI) and sentence comprehension (SC). The experiment was conducted with 171 children between 11 and 13 years old in ecologically-valid conditions (collective presentation in real, reverberating classrooms). Two standardized tests were used to assess SI and SC. The two tasks were presented in three listening conditions: quiet; traffic noise; and classroom noise (non-intelligible noise with the same spectrum and temporal envelope of speech, plus typical classroom sound events). Both task performance accuracy and listening effort were considered in the analyses, the latter tracked by recording the response time (RT) using a single-task paradigm. Classroom noise was found to have the worst effect on both tasks (worsening task performance accuracy and slowing RTs), due to its spectro-temporal characteristics. A developmental effect was seen in the range of ages (11–13 years), which depended on the task and listening condition. Gender effects were also seen in both tasks, girls being more accurate and quicker to respond in most listening conditions. A significant interaction emerged between type of noise, age and task, indicating that classroom noise had a greater impact on RTs for SI than for SC. Overall, these results indicate that, for 11- to 13-year-old children, performance in SI and SC tasks is influenced by aspects relating to both the sound environment and the listener (age, gender). The presence of significant interactions between these factors and the type of task suggests that the acoustic conditions that guarantee optimal SI might not be equally adequate for SC. Our findings have implications for the development of standard requirements for the acoustic design of classrooms.

## Introduction

Oral communication in classrooms is a complex phenomenon involving different types of speech material (from simple commands to complex lectures) and speaker-listener interactions (e.g., teacher to class, one-to-one during group work, one to small group, etc.). While these two factors may combine in various ways, giving rise to different communication scenarios, all of the currently-used standards for classroom acoustics are only conceived to guarantee speech intelligibility (SI). The standards provide for limits in terms of acoustic indicators, which are designed to account for the separate and/or joint effects of background noise and reverberation on speech reception (e.g., the Speech Transmission Index of the International Electrotechnical Commission, 2011). Unfortunately, SI is on the surface of the levels of representation involved in verbal processing (Hustad, 2008), and it mainly provides information about the correct reception of the acoustic-phonetic cues in a message. Differently, communication during lessons requires a higher level of language processing. It relies on messages with variable syntactic forms, and on lexical, semantic and contextual information, and listeners are expected not only to understand the content, but also to integrate it with previously acquired experience and knowledge.

The testing of listening comprehension in adult and pediatric populations has been the object of several publications. Specific tests have been developed, based on listening to text passages and answering content questions (Valente et al., 2012; Sullivan et al., 2015; Rudner et al., 2018; von Lochow et al., 2018), or on implementing oral instructions (Klatte et al., 2010a). The tasks presented in such studies are similar to tasks that students perform in their everyday life, and are consequently ecologically valid, but their inherent complexity can make them difficult to administer routinely for the assessment of classroom acoustics.

To improve on assessments based on SI alone, a viable alternative to listening comprehension is to consider sentence comprehension (SC). This approach provides information on levels of language processing beyond speech reception because auditory, syntactic, contextual, and semantic information can be manipulated in a simple and scalable manner. For instance, Uslar et al. (2013) described how linguistic complexity could be modulated to improve the audiological matrix sentence test for adults (Wagener et al., 1999), and gain information on the usage of their cognitive capacity while listening in noise. It is generally assumed that the more the extraction of meaning from the speech signal is elaborate, the greater the burden on the top–down cognitive resources of the listener (Downs and Crum, 1978), leaving less cognitive capacity left over for higher-level speech processing (Rudner and Lunner, 2014). Increasing the linguistic difficulty of sentences, or chaining the sentences together would thus help to clarify the speech processing needs in classrooms, adding to the information provided by the basic SI results. Comparisons between the two tasks (SI and SC) have not been conducted systematically, whereas some results are available for comparisons between SI and certain more complex listening comprehension tasks. For instance, Fontan et al. (2015) tested young adults and, using a task that involved commands to move objects, they retrieved transcripts of instructions for SI and also monitored subsequent actions. When the authors compared the scores for SI and comprehension, they found a modest correlation between the two tasks (*r* = 0.35), and concluded that SI was a poor predictor of comprehension in real communication settings. Klatte et al. (2010a) compared SI (word-to-picture matching) and comprehension (execution of oral instructions) in 7- and 9-year-old children, using classroom noise (typical classroom sounds without speech) and background speech as maskers. They found that classroom noise had a stronger effect on SI, but background speech was more harmful for comprehension.

Overall, the literature points to a weak relationship between task performance accuracy in SI and comprehension tasks for normally-hearing listeners. Fontan et al. (2017) points out that intelligibility and comprehension measures might be considered as complementary, providing information on different aspects of speech communication. Exploring the effects of noise and reverberation on both tasks could therefore facilitate the development of effective tools for controlling the sound environment in the classroom, considering at once speech signal transmission and communicative performance.

Several explanations have been advanced for the specific impact of noise and reverberation on verbal task outcomes in classrooms. In particular, the way noise interferes with speech depends not only on the level of noise, but also on its spectro-temporal characteristics. The adverse effect of a background noise may originate from either energetic or informational masking (Mattys et al., 2012). In the former case, speech and masker overlap in time and frequency in such a way that portions of the signal are no longer audible (Brungart, 2001). This form of masking is supposed to take place at the level of the auditory periphery and the recognition process relies mainly on stream segregation and selective attention. Adult listeners experience an advantage in speech reception for temporally fluctuating maskers compared with steady-state maskers presented at the same noise level. This so-called “masking release” originates from a combination of factors (see Füllgrabe et al., 2006 for a complete review), including dip listening, or the listener’s ability to exploit short periods with high signal-to-noise ratios (SNR), when the fluctuating noise was lowest, to detect speech cues. The fluctuations in the background noise may also interfere with the temporal fluctuations in the speech, giving raise to the modulation masking, which counterbalances dip listening. Informational masking is believed to have consequences on speech recognition that go beyond its energetic effect, such as attentional capture, semantic interference, and increased cognitive load. Background speech with intelligible and meaningful content may result in informational masking, as its interference directly affects working memory by competing with the target speech. Non-speech sounds may produce informational masking as well. As Klatte et al. (2010b) pointed out, however, the various effects of non-speech sound cannot be explained by a single mechanism. Depending on its characteristics, a sound may have a changing state effect (e.g., when the sound consists of distinct auditory objects that vary consecutively; see Hughes and Jones, 2001), or an attentional capture effect (e.g., salient, unexpected, or deviant auditory events; see Klatte et al., 2013), or a mixture of both.

With specific reference to the effect of background noise on children in classrooms, Klatte et al. (2007) found higher-level cognitive processing more affected by unintelligible background speech than by traffic noise, when the two noises were presented at the same level; the authors related the difference to the changing-state characteristics of the background speech. Dockrell and Shield (2006) compared quiet, babble, and babble plus environmental noise conditions, testing 7- to 8-year-old children with verbal tasks (reading and spelling). They found the children’s performance accuracy negatively affected by classroom babble, and suggested that verbal tasks involving working memory processes are more vulnerable to the interference of concurrent speech.

Like background noise, reverberation in the classroom can also increase the speech processing burden. Normative values have been established for optimal reverberation times, which depend on the classroom’s volume and the use made of the space (Deutsche Institut für Normung, 2016). Several studies have demonstrated the importance of assessing the combined effects of noise and reverberation in classrooms, given the greater effect of adverse listening conditions on children than on adults. Prior research indicated that speech recognition in noisy and reverberating conditions improves with age (Neuman et al., 2010) and consonant identification does not reach adult-like performance accuracy until the age of 14 years (Johnson, 2000). Children are also more easily distracted by auditory events due to their less robust and less developed attentional abilities (Klatte et al., 2013; Meinhardt-Injac et al., 2014), and their performance accuracy deteriorates the most in speech-in-speech tasks (with competing speech from two talkers, see Corbin et al., 2016). Masking release is also more limited in children (up to 13–14 years old) than in adults, when a speech-shaped, amplitude-modulated noise is presented in reverberating conditions (Wróblewski et al., 2012). Leibold (2017) suggested that this latter finding might indicate that children are not as good as adults at glimpsing speech in fluctuating noise.

Most of the available data about children’s speech processing in the classroom are based on their accuracy in completing tasks, while few studies have also considered their response times (RTs) measured using a single-task paradigm in order to judge their listening effort. In this context, RT is intended as a measure of speed of processing, and provides information on the amount of cognitive capacity allocated to processing the auditory signal (Pichora-Fuller et al., 2016). Several published studies indicate that, like other measures of listening effort, changes in RT may mirror changes in task performance accuracy (e.g., Lewis et al., 2016; McGarrigle et al., 2019), but they may also occur when accuracy is at or near ceiling level (Hällgren et al., 2001), or kept constant (Uslar et al., 2013; Sahlén et al., 2017). On the whole, the literature supports the hypothesis that accuracy and listening effort might represent two different constructs in the general frame of speech processing: the two measures are not always related (Wendt et al., 2018), and factors affecting task performance accuracy do not affect listening effort to the same degree (Picou et al., 2016). Measures of listening effort are generally considered valuable to complement traditional speech-in-noise tests, and provide additional information beyond task performance accuracy.

With specific reference to the use of RTs in the pediatric population, Lewis et al. (2016) used verbal RTs as a proxy for listening effort in a study on normally-hearing children from 5 to 12 years old, and children with hearing loss. The children with a normal hearing function had longer RTs with decreasing SNR. These results were confirmed by McGarrigle et al. (2019), who also found that verbal RTs were more effective than visual, dual-task RTs for children 6 to 13 years old. Prodi et al. (2013) combined SI with RTs for 8- to 11-year-olds. This method enabled a ranking of the interference of different types of noise, and revealed changes in the balance between signal-driven and knowledge-driven processes. SI improved and RTs decreased with increasing age, but the changes in the two metrics followed different patterns. The increase in task performance accuracy with older age came first, and it was only after accuracy reached the ceiling that a decrease in RTs with increasing age became apparent.

The general mechanisms governing the effects of noise and reverberation on speech reception are sufficiently well-known and documented for primary school children, but there is a need to extend what we know to less well-researched age ranges, such as 11- to 13-year-olds. The ability to hear and understand speech in adverse conditions matures during childhood, but the age at which an adult-like performance is reached depends on the nature of the background noise (Leibold, 2017). In complex acoustic environments, with non-stationary noises and reverberation, 13- to 14-year-olds perform less well than adults (Wróblewski et al., 2012): this gives the impression that children up to this age might continue to be at a particular disadvantage when listening in adverse conditions. In addition, the comparison between performance accuracy results in SI and SC has been pursued for adults (Hustad, 2008; Fontan et al., 2015), and for children aged 7 and 9 years (Klatte et al., 2010a), but no investigations have been conducted on older school-age children. A better understanding of how noise, age and task may interact would be valuable when tailoring classroom acoustics to optimize learning performance and reduce listening effort.

Previous studies on developmental changes in speech processing ability in the classroom have also considered the issue of gender differences. Ross et al. (2015) tested a group of typically-developing children from 5 to 17 years old over a fairly wide range of SNRs using a speech recognition task with isolated, monosyllabic words. They found that females performed better than their male peers in both audio-only and audio-visual presentation modes. When Boman (2004) investigated the interaction between gender and noise in 13-to 14-year-olds using episodic and semantic memory tasks, girls had a better recall performance than boys, and this finding was consistent across different verbal materials. No interaction emerged between gender and noise as the presence of noise affected the boys’ and girls’ performance to the same degree. Listening effort has only been considered in relation to gender in the case of voice quality deterioration, and for 8-year-olds (Sahlén et al., 2017). In the study by Sahlén et al. (2017), a SC test was administered in multi-talker babble noise and the RTs for listening conditions in which girls and boys performed equally well were considered (Lyberg-Åhlander et al., 2015). Unlike task performance accuracy, latencies were longer for girls than for boys. Considering these results together, it is unclear whether the girls’ better performance accuracy – reported by Boman (2004) and Ross et al. (2015) – coincided with slower processing times, or whether the findings of Sahlén et al. (2017) concerning listening effort related to the particular testing conditions (dysphonic voice) or to differences in the strategies used by girls and boys to solve the task.

The present work reports on SI and SC tasks presented in real reverberating classrooms. The participants consisted of a fairly large group of children 11 to 13 years old, who collectively performed the tasks in three listening conditions: quiet; traffic noise; and classroom noise (speech-like noise plus typical classroom sounds). Both tasks were presented in a closed-set format, using personal portable devices (tablets). Two outcome measures were considered (task performance accuracy and RTs), and used to obtain a comprehensive view of the speech processing phenomenon. RTs were used as a behavioral measure to quantify listening effort, assuming that slower RTs reflect a greater listening effort.

The tasks were presented to 11- to 13-year-old children in their classrooms. The research questions addressed were as follows:

(1) Depending on the task and the type of noise, what is the interplay between task performance accuracy and listening effort when children have to cope with noise? Does age have any effect?
(2) Are there gender-related differences in SC and SI task performance? Do these differences regard task accuracy alone, or listening effort as well?
(3) When both SI and SC are evaluated under the same acoustic conditions, does age and type of noise similarly influence performance accuracy and listening effort in the two task?

## Materials and Methods

### Description of the Classrooms

The experiment took place in the first half of the school year (November–December, 2018) at two schools in Ferrara, Italy. One classroom was chosen at each school for use as a laboratory during the test sessions. Both classrooms were box-shaped, with similar volumes (152 and 155 m^3^), and dimensions (7.3 m long × 7.0 m wide × 3.0 m high; and 8.3 m × 6.0 m × 3.1 m). During the experiments, the classrooms were set up as for regular lessons, with wooden desks and chairs arranged in rows and facing the teacher’s desk.

Only one of the classrooms had sound-absorbing ceiling tiles, so the other classroom was temporarily fitted with sound-absorbing polyester fiber blankets to balance the acoustic conditions in the two rooms. This temporary solution ensured the same reverberation times across the octave band frequencies in both classrooms: the T_mid_ (average reverberation time for the octave bands 500–2000 Hz) in occupied conditions was 0.68 and 0.69 s respectively. At the time of testing, the number of pupils sitting in the classrooms ranged between 14 and 23, depending on the number of students belonging to each class.

### Participants

A total of 171 pupils between 11 and 13 years old belonging to nine different classes at two different schools took part in the study. The school administrations gave their permission for the study. The study was approved by the Ethics Committee of the University of Padova (Italy). Written informed parental consent was obtained prior to any testing.

After the experiment, the teachers provided details about children with intellectual disabilities and hearing impairments (as certified by the National Healthcare System). There were six such children (three at each school), who were excluded from the subsequent data analysis. The results for another six children were also omitted from the analysis due to: the baseline comprehension score in four cases (two children did not complete the assessment, and two scored lower than the threshold); and an extremely low performance in the SI task (quiet condition) in two, indicating that the children misunderstood the instructions.

The final sample of participants is detailed in Table 1.

**TABLE 1:** Characteristics of the children participating in the study.

**Age group**	**No. of participants**	**% Male/female**	**Age [M (SD); range]**
11 years	53	49/51	11.0 (0.3); 10–12
12 years	49	53/47	11.9 (0.2); 11–12
13 years	57	58/42	12.9 (0.3); 12–13
All	159	53/47	12.0 (0.9); 10–13

### Reading Comprehension Assessment

Before conducting the experiment, pupils were screened for comprehension problems that could influence the study outcomes. Given the association between listening and reading comprehension (Wolf et al., 2019), a measure of reading comprehension was used for this purpose.

Students were collectively presented with the measures in a quiet condition. The assessment took place nearly 1 week after presenting the SI and SC tasks. A standardized reading comprehension test based on the participants’ school grade was administered (derived from Cornoldi et al., 2017). Participants were given text passages to read silently. Then they had to answer 15 multiple-choice questions without any time constraints, and could refer back to the passage while answering. Cronbach’s alpha was higher than 0.71 for all tasks, indicating an acceptable internal consistency.

For each age group, differences between classes and genders were examined with reference to the reading comprehension assessment. No significant differences emerged between the genders, whereas there were significant differences between the classes (see Table 2).

**TABLE 2:** Significance tests for the reading comprehension task, by class (three for each age group) and gender: Mann–Whitney’s *U*-test on gender, Kruskal–Wallis test for classes.

**Age group**	**Class**	**Gender**
11 years	χ^2^(2) = 7.15, *p* = 0.03	W = 372, *p* = 0.92
12 years	χ^2^(2) = 7.42, *p* = 0.02	W = 450, *p* = 0.21
13 years	χ^2^(2) = 13.42, *p* < 0.001	W = 463, *p* = 0.91

### Speech Intelligibility Task – Stimuli, Procedure, and Dependent Variables

Speech intelligibility was assessed with the Matrix Sentence Test in the Italian language (ITAmatrix, see Puglisi et al., 2015). This test is based on five-word sentences, with a fixed syntactic structure but no semantic predictability (e.g., *Sofia compra poche scatole rosse* [Sophie buys few red boxes]). Each sentence is generated from a 10 × 5 base-word matrix, with 10 options for each word in the sentence.

Digital recordings of the sentences were acquired by agreement with the producer, Hoertech GmbH. The average sentence duration was 2.3 s. Three lists of 16 sentences were created for the experiment, plus four additional sentences for the training phase.

For each trial comprising the task, participants were presented aurally with the playback of a sentence. After the audio offset, the base-word matrix was displayed on the tablets and participants had to select the words they had heard in serial order (i.e., in the same order in which the words were played back). It was impossible to change a response once the selection had been made. Participants were allowed a maximum of 15 s to select the five words.

The score (right/wrong) for each word comprising the sentence was recorded and used to evaluate the SI score, defined as the percentage of words correctly recognized in the sentence. RTs (i.e., the time elapsing between the end of the waveform of the last word in the sentence heard and the selection of the first word on the tablet) was automatically recorded for each participant and trial.

### Sentence Comprehension Task – Stimuli, Procedure, and Dependent Variables

Sentence comprehension was examined using the COMPRENDO Test (Cecchetto et al., 2012), which is designed to assess comprehension of a series of sentences in the Italian language. The sentences differ in their syntactic complexity: transitive active sentences (e.g., *La mamma sta inseguendo il bambino* [The mother is chasing the child]), dative sentences (e.g., *Il papà dà il latte alla bambina* [The father gives milk to the little girl]), active sentences with two objects (e.g., *Il bambino insegue il cane e il gatto* [The child chases the dog and the cat]), coordination between active sentences (e.g., *Il bambino guarda il gatto e la mamma accarezza il cane* [The child looks at the cat and the mother strokes the dog]), sentences with subject relative clauses (e.g., *Il bambino che saluta il nonno guarda la televisione* [The child who greets his grandfather is watching television]), and sentences with object relative clauses (e.g., *Il nonno spinge il cane che morde il gatto* [The grandfather pushes the dog that is biting the cat]). All the sentences (10 for each type) were generated using 20 nouns and 20 verbs that were easy to understand and in very common use. Material selection occurred in two phases. In the first phase, 200 nouns and 200 verbs with higher frequency were selected from the Laudanna et al. (1995) database. In the second phase, a group comprised by one psychologist, one speech-language pathologist, and one neuropsychologist, selected the nouns and verbs to use for the material of the study among the 400 words obtained in phase one.

The sentences were recorded in a silent room by a native Italian, female, adult speaker. A B&K Type 4189 1/2 inch microphone was placed about 20 cm from the speaker’s mouth and routed to a B&K Type 5935 signal conditioner. The digital recordings had a 16-bit resolution and a 44100 Hz sampling rate. The sentences were digitally filtered to match the long-term spectrum of the female speaker in the ITAMatrix. The sentences lasted a mean 3.4 s. Three different lists of 16 sentences each were prepared using a pseudo-randomized procedure to ensure that the same number of sentences was presented for each level of syntactic complexity in each list.

**FIGURE 1:**
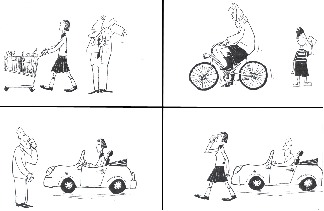
Example of a trial in the sentence comprehension (SC) task, with the four images displayed on the individual tablets. The spoken sentence was “The man drives the car and the woman drinks the milk.”

During the experimental session, the sentences were aurally presented to participants. After the audio offset of each sentence, four images appeared (one for each quadrant on the screen), and participants were asked to touch the image that properly described the sentence they had just heard (Figure 1). RTs and accuracy were recorded for each sentence. A time-out of 12 s was set for selecting an answer.

### Background Noises and Listening Conditions

Three listening conditions were considered in the study: quiet, traffic noise, and classroom noise. For the traffic noise, recordings were obtained alongside a busy road in conditions of dense traffic, including cars and trucks. The recordings were spectrally filtered to account for the sound insulation properties of a typical building façade. For the classroom noise, Italian phrases spoken by a native female speaker were processed according to the established ICRA procedure (Dreschler et al., 2001). The resulting signal had speech-like fluctuations and the same spectrum as Italian speech, but was not intelligible. Sound events typical of a busy classroom were added to this signal by digital mixing (e.g., a pen rolling off a desk onto the floor, chairs scraping, pages being turned over in a book).

**FIGURE 2:**
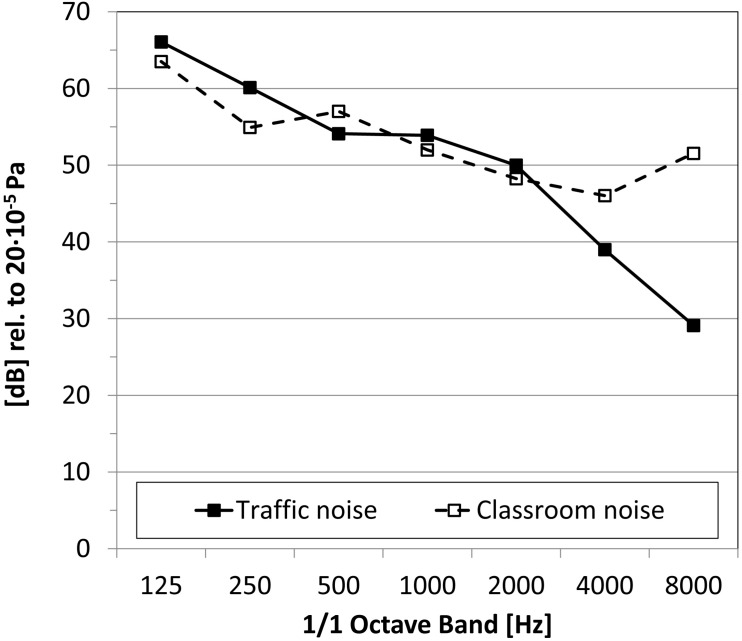
Octave band, long-term average spectra of background noises. The overall A-weighted level is set to 60 dB(A).

**FIGURE 3:**
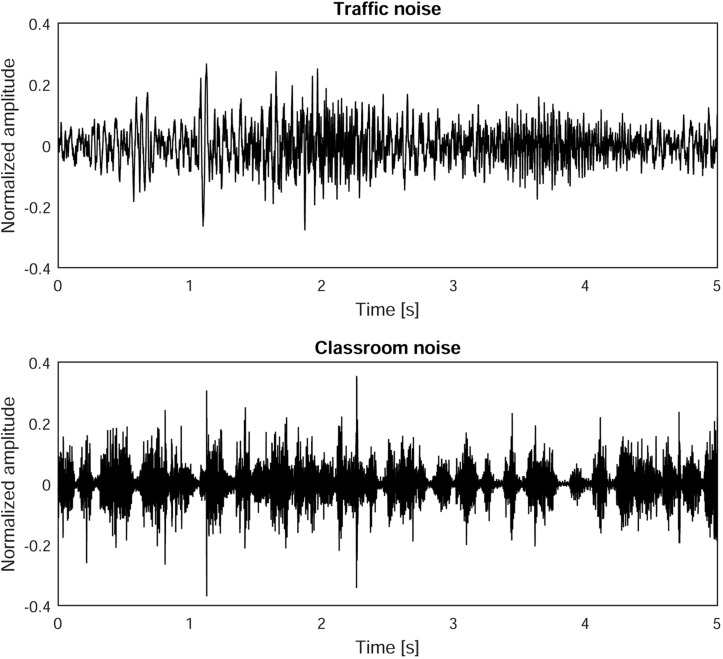
Temporal pattern of background noises used in the experiment.

The long-term averaged spectral characteristics of the two types of background noise are shown in Figure 2. The classroom noise had typical speech-like components plus higher frequencies due to sounds common in classrooms being mixed with the babble. The traffic noise had a more balanced frequency trend up to 2 kHz, then sloped down. Figure 3 shows the temporal pattern of the two types of background noise, recorded in anechoic conditions. The classroom noise had faster fluctuations, showing shallow depths and sparse peaks, whereas the traffic noise had slower fluctuations. The amount of fluctuation over time of the noise levels was also qualified using the difference in the percentile sound levels (i.e., L_A,__10_ – L_A,__90_). By definition the L_A,__10_ value is the level exceeded for 10% of the measurement time, and takes into account the presence of peaks of noise. L_A,__90_ is the level exceeded for 90% of the measurement time, and accounts for the residual noise level. The difference between the two percentile sound levels gives an indication of the stationarity of the noise: the difference is low for stationary noise, while it increases for noises with temporal fluctuations. In anechoic conditions the difference was 7.0 and 8.1 dB for the traffic and classroom noise, respectively.

For the test sessions, two loudspeakers were placed inside the classroom. A Gras 44AB mouth simulator used to deliver the speech signals was placed close to the teacher’s desk, at a height of 1.5 m (assumed as the height of a standing teacher’s mouth), and it was oriented toward the audience. The background noises were played back with a Look Line D303 omnidirectional source placed on the floor near the corner of the room closest to the teacher’s desk.

In all listening conditions, the speech signal was fixed to a level of 63 dB(A), measured at 1 m in front of the mouth simulator. This corresponds to a speaker talking with a vocal effort qualified as intermediate between “normal” and “raised” (International Organization of Standardization, 2003). This choice of sound pressure level was based on the findings of Bottalico and Astolfi (2012), who measured the average vocal effort of female teachers during the working day, finding a mean sound pressure level of 62.1 dB(A) at 1 m from the speaker’s mouth.

In the quiet condition the speech signals were presented against the background ambient noise of the classroom, which consisted of noises coming from adjacent classrooms, where students were engaging in quiet activities. When the tasks were presented in traffic or classroom noise, the playback level was fixed at 60 dB(A), measured as the spatial average over four positions defined in the seating area. This value was chosen to represent a typical level measured in occupied classrooms during lessons, in accordance with the report from Shield et al. (2015), who found that the levels measured during lessons in secondary schools vary between 50 and 70 dB(A).

An objective description of the acoustic conditions experienced by the audience during the test session was obtained with the Speech Transmission Index (STI; International Electrotechnical Commission, 2011). The metric quantifies the loss of modulation of the speech signal during its transmission from the source to the receiver, accounting for the adverse effects of background noise and reverberation. The STI is in the range of [0; 1], the upper limit corresponding to perfect speech transmission.

**FIGURE 4:**
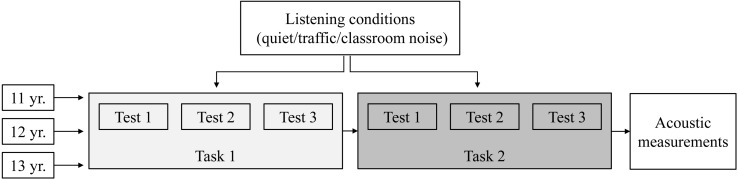
The experimental design for the three age groups (11, 12, and 13 years old). Three tests were presented for each task [speech intelligibility (SI) and sentence comprehension], one for each listening condition. The order of the two tasks and the order of the listening conditions were balanced across classes in each age group.

All measurements were obtained using a B&K type 4189 1/2 inch microphone plus a B&K Type 4231 calibrator, connected to a B&K Type 5935 signal conditioner and a RME Fireface UC full-duplex sound card. The impulse responses and sound pressure levels were measured for each class participating in the study. These measurements were obtained at the end of the experimental session, with the classroom still occupied (see Figure 4). Four receiver positions were defined in each classroom, evenly distributed in the area where the students were seated during the experiment, at representative seats. Each microphone was placed at least 1.00 m away from the walls and at a height of 1.20 m (assumed as the height of a student’s ears when seated). Care was taken to ensure that the microphone was not shielded by the head or body of the student seated in the row ahead. The students were asked to remain quiet during the measurements.

For each class, the spatial deviation of the acoustic parameters (T_30_, sound levels, STI) was considered first. The values measured at the four receivers always differed by a quantity smaller than the corresponding “just noticeable difference” (JND): 5% for the reverberation time, 1 dB for the sound pressure level (International Organization of Standardization, 2009), and 0.03 for the STI (Bradley et al., 1999). This result demonstrates a rather uniform spatial behavior at the seating positions in the classroom, in line with previous studies considering classrooms with sizes comparable to ours (Astolfi et al., 2008, 2012; Prodi et al., 2013). It should be noted that all seating positions were located outside the critical radius (r_c_) of the classrooms (i.e., the distance from a sound source at which the level of the direct sound equals the reflected sound level), which was 1.5 m for both classrooms. The seating position closest to the speech source (in the first row of desks, directly facing the source) was 2.10 m from the speech source in one room, and 1.95 m in the other. In the reverberant field, which takes over outside r_c_, the sound field is primarily driven by the multiple reflections from the room boundaries. The small dimensions of the classrooms and the presence of a reverberant sound field thus meant that the acoustic parameters had very similar values (no more than 1 JND) in the various seating positions. The spatial uniformity of the acoustic parameters in the two rooms is a guarantee that, for these classrooms and seating areas, the listening conditions were equivalent in the different seating positions.

Then the deviation in the acoustic parameters between different groups of students was considered. The differences in the acoustic parameters between repetitions over the classes were always smaller than 1 JND, so the final values for the acoustic parameters in the classrooms were averaged across the repetitions (Table 3).

**TABLE 3:** Listening conditions in the two classrooms (A, B) during the experiment: reverberation time T_mid_ (averaged across 500–2000 Hz octave bands), A-weighted sound pressure level L_A,eq_ dB(A), Speech Transmission Index (STI).

**Acoustic parameter**	**Classroom A**	**Classroom B**
Reverberation time T_mid_ [s]	0.68 (0.66; 0.69)	0.69 (0.68; 0.71)
Speech: L_A,eq_ dB(A)	60.4 (59.9; 60.6)	59.5 (59.2; 59.9)
Quiet: L_A,eq_ dB(A)	43.3 (43.2; 43.4)	40.5 (39.7; 40.6)
Traffic noise: L_A,eq_ dB(A)	60.9 (60.7; 61.2)	59.9 (59.4; 60.1)
Classroom noise: L_A,eq_ dB(A)	60.5 (60.3; 60.6)	60.1 (59.6; 60.4)
Quiet: STI	0.64 (0.63; 0.64)	0.65 (0.64; 0.65)
Traffic noise: STI	0.46 (0.44; 0.46)	0.48 (0.47; 0.49)
Classroom noise: STI	0.40 (0.39; 0.40)	0.39 (0.39; 0.40)

*All measurements were taken with the rooms occupied. The reported values are spatial averages across four positions in the audience, and across repetitions over the classes. In brackets, maximum and minimum values measured with different groups of children.*

It is worth emphasizing that the differences between the listening conditions in the two classrooms were always smaller than the JND for all the acoustic parameters, except for the sound pressure level in the quiet condition. So, for the purpose of our study, the two rooms can be considered as equivalent from the acoustic perception standpoint (Bradley et al., 1999; Postma and Katz, 2016).

### Procedures

Participants completed the experiment in groups consisting of whole classes, which took turns in the laboratory classroom over the course of their morning lessons. The numbers of students in each class ranged between 14 and 23. The test session (including the presentation of the task and the acoustic measurements) took 1 h for each class.

At the start of the test session, each child was given a tablet, and was randomly assigned to a seat. Then participants were instructed to enter their age in years and the identification code they found on their desk on their tablets. Using this code ensured that listening positions, test devices and participants were matched correctly, and also ensured anonymity when handling the results. Each child was asked to remember their code and write it on the booklet used for their reading comprehension assessment, which took place on the following days. The same teacher supervised both sessions and ensured the correct matching between participants and codes.

Before starting the experiment, participants were briefly informed about the aim of the study. Then the two tasks were performed, one after the other. To avoid order and fatigue effects, the order of the two tasks was balanced across the classes in each age group. Before each task, participants were given verbal instructions and familiarized with the task and the data collection system by presenting a set of four trials in quiet conditions. Then they completed three tests (one for each listening condition). The listening conditions were balanced across the classes in each age group. The test lists were pseudo-randomized to avoid coupling the same test list with the same listening condition. An outline of the experimental design is shown in Figure 4.

During the tests the background noises (traffic or classrooms noise) started approximately 1 s before the target sentence and ended simultaneously with the speech signal. In the quiet condition, an acoustic signal (brief pure tone at 500 Hz) was played back 1 s before the spoken sentence. Each experimental trial was time-limited (to 12 or 15 s, depending on the task). It was only once all participants had responded or reached the time-out that the next target sentence was automatically played back.

Participants were instructed to pay attention to the task, and to respond as accurately as possible. They were not told that RT data would be acquired, nor were they urged to respond as quickly as possible.

The whole experiment was managed by using a wireless test bench (Prodi et al., 2013), based on a server application which simultaneously controlled the audio playback, the presentation of the base-matrix/images on the tablets, and the data collection.

### Data Analysis

Two outcome variables were considered for each task: task performance accuracy and RT.

Before any analysis, data points where technical errors occurred (e.g., loss of the connection between the server and a tablet) were removed from the databases: altogether, 1.2% of the SI trials and 0.7% of the SC trials were discarded for such reasons. Data points corresponding to trials for which the time-out was reached were also removed: this applied to 5.9% of the trials in the SI task and 0.7% of the trials in the SC task.

The statistical analysis was performed using generalized linear mixed-effects models (GLMMs). This statistical method was chosen because it can be used to deal with non-independent individual responses (repeated-measures design) and data for which the normality assumption is not met (Lo and Andrews, 2015; Gordon, 2019). A binomial distribution was adopted in the statistical model for accuracy data, which are bound within the [0; 1] interval, while a Gamma distribution with a log link function was used for the raw RT data.

To analyze each outcome variable in each task, four separate GLMMs were set up (2 tasks × 2 outcome variables). The fixed effects considered in the models were: listening condition (quiet, traffic, classroom noise); age (11, 12, 13 years); gender (male, female); and all two- and three-way interactions. Because the participants differed significantly in their baseline scores (see Table 2), the score in the reading comprehension test was included in the models as a covariate. In all the models, the participant variable was included as a random intercept. The listening condition within-subject factor was also included in the random effects as a random slope. The GLMM thus allowed for the listening condition to have a different effect for each participant.

Then, a second analysis was run to compare the tasks directly in the different listening conditions. This was done by setting up a linear mixed-effects model (LMM), with the relative change in RTs as the outcome variable. The quantity was defined by the ratio of the median RT in noise to the median RT in quiet for each task. The distribution of the raw RTs across the trials was skewed, so the median of the 16 trials was calculated for each combination of participant, listening condition and task, and this was used to calculate the ratio. The resulting quantity reflects the amount of change in processing time due to the addition of background noise. The quiet condition took a value of one for all participant-task combinations, while higher values indicated longer RTs compared with the quiet condition. The fixed effects considered in the LMM were: listening condition (traffic and classroom noise; as quiet was assigned a value of one by definition, it was not included in the model); age (11, 12, 13 years); gender (male, female); task (speech intelligibility, sentence comprehension); the two-way interactions including task and listening condition, and the three-way interaction between age, listening condition and task. The score in the reading comprehension task was added to the models as a covariate. A random intercept (participant) and two random slopes (the within-participant variables listening condition and task) were also specified.

Values for the GLMMs and LMM were obtained using likelihood ratio tests. The consistency of the models was investigated by checking their assumptions, which meant controlling the normality of the random effect terms and the residuals, as suggested by Everitt and Hothorn (2010).

The analysis was conducted using the *R* software (R Core Team, 2017) and the *lme4* package (Bates et al., 2015). *Post hoc* pairwise comparisons were performed using least-squares means tests with the *lsmeans* package (Lenth, 2016). In the case of multiple comparisons, the Bonferroni method was applied to adjust the *p*-values. The statistical significance threshold was set at 0.05.

## Results

### Speech Intelligibility: Accuracy

**FIGURE 5:**
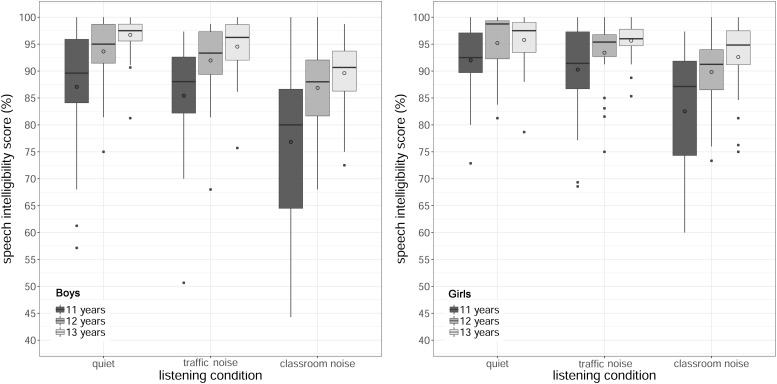
Boxplots of accuracy in the speech intelligibility task by age and listening condition, for boys **(left)** and girls **(right)**. The length of the box corresponds to the interquartile range of the data distributions; the central, bold line is the median value, and the white circle is the mean; 99% of the data fall within the whiskers. Outliers are shown as black circles outside the whiskers.

Figure 5 shows the SI scores by age and listening condition, for boys and girls. The analysis revealed a statistically significant main effect of listening condition [χ^2^(2) = 189.23, *p* < 0.001]. *Post hoc* tests comparing listening conditions collapsed across age and gender revealed that task performance accuracy was significantly better in quiet than in noisy conditions (quiet > traffic noise, *z* = 4.11, *p* < 0.001; quiet > classroom noise, *z* = 11.82, *p* < 0.001), and in classroom noise than in traffic noise (traffic noise > classroom noise, *z* = 10.25, *p* < 0.001). The SI scores were 1.6% higher in quiet than in traffic noise, and 5.5% higher in traffic noise than in classroom noise.

The analysis also revealed a significant main effect of age [χ^2^(2) = 56.42, *p* < 0.001]. *Post hoc* tests with the results collapsed across listening condition and gender showed a worse performance accuracy for the youngest children than for the others (11 < 12 years, *z* = −5.66, *p* < 0.001; 11 < 13 years, *z* = −6.88, *p* < 0.001). The mean results were 85.7% (*SD* = 11.7%), 91.8% (*SD* = 7.3%) and 94.1% (*SD* = 6.0%) for 11-, 12-, and 13-year-olds, respectively.

Finally, the analysis showed a significant main effect of gender [χ^2^(1) = 56.42, *p* < 0.001], with girls performing significantly better (*M* = 91.8%, *SD* = 8.3%) than boys (*M* = 89.6%, *SD* = 10.1%). The main effect of the reading comprehension score [χ^2^(2) = 20.72, *p* < 0.001] was significant as well.

There were no interactions between listening condition and age (*p* = 0.84), between listening condition and gender (*p* = 0.59), or between age and gender (*p* = 0.84). There was also no significant three-way interaction between listening condition, age and gender (*p* = 0.12).

### Speech Intelligibility: RTs

**FIGURE 6:**
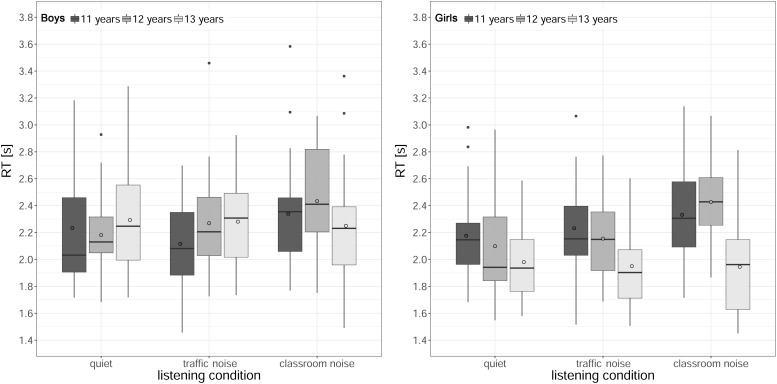
Boxplots of response times (RTs) in the speech intelligibility task by age and listening condition, for boys **(left)** and girls **(right)**. The length of the box corresponds to the interquartile range of the data distribution; the central, bold line is the median value, and the white circle is the mean; 99% of the data fall within the whiskers. Outliers are shown as black circles outside the whiskers.

Figure 6 shows the RTs (median across the trials) for each listening condition and age, for boys and girls. The analysis revealed a significant main effect of listening condition [χ^2^(2) = 25.41, *p* < 0.001], a main effect of age [χ^2^(2) = 6.61, *p* < 0.001], and a main effect of gender [χ^2^(1) = 8.66, *p* = 0.003]. The two-way interactions between listening condition and age [χ^2^(2) = 25.41, *p* < 0.001], and between age and gender [χ^2^(2) = 25.41, *p* < 0.001] were significant as well. The main effect of the baseline comprehension score and the remaining two- and three-way interactions were not significant (all *p*s > 0.15).

The significant interaction between listening condition and age was considered first, with data collapsed across genders. When the effect of noise was analyzed for each age group, the RTs for the 11- and 12-year-olds were significantly slower in classroom noise than in quiet or traffic noise conditions, while there was no difference between quiet and traffic noise (11 years: quiet < classroom noise, *z* = −3.20, *p* = 0.004, ΔRT = 130 ms; traffic noise < classroom noise, *z* = −2.74, *p* = 0.018, ΔRT = 160 ms; 12 years: quiet < classroom noise, *z* = −4.85, *p* < 0.001, ΔRT = 288 ms; traffic noise < classroom noise, *z* = −3.47, *p* = 0.002, ΔRT = 214 ms). For the 13-year-olds, on the other hand, there was no difference between listening conditions. When the effect of age was analyzed for each listening condition, pairwise comparisons revealed that RTs only differed across ages in classroom noise, being faster for the oldest students (11 > 13 years, *z* = 3.29, *p* = 0.003, ΔRT = 213 ms; 12 > 13 years, *z* = 3.45, *p* = 0.002, ΔRT = 308 ms). When the interaction between age and gender was analyzed, with data collapsed across listening conditions, *post hoc* tests indicated that it was only among the 13-year-olds that RTs for girls were a mean 316 ms faster than for boys (girls < boys, *z* = −3.97, *p* < 0.001).

### Sentence Comprehension

Table 4 shows SC performance accuracy as the percentage of correct answers across ages for the three listening conditions. The results showed a strong ceiling effect, with most pupils achieving or coming close to the highest score in all listening condition. Given this ceiling effect, and the small degree of variance in accuracy in the SC task, only the corresponding RTs were included in the analysis.

**TABLE 4:** Mean percentage of correct answers and standard deviations (in brackets) in the sentence comprehension task, in the three listening conditions and age groups.

	**Listening condition**		
**Age group**	**Quiet**	**Traffic noise**	**Classroom noise**
11 years	95.0 (6.9)	95.3 (6.4)	92.2 (7.5)
12 years	95.7 (4.9)	94.4 (5.5)	93.4 (7.8)
13 years	96.5 (5.7)	95.9 (5.0)	94.7 (6.4)

**FIGURE 7:**
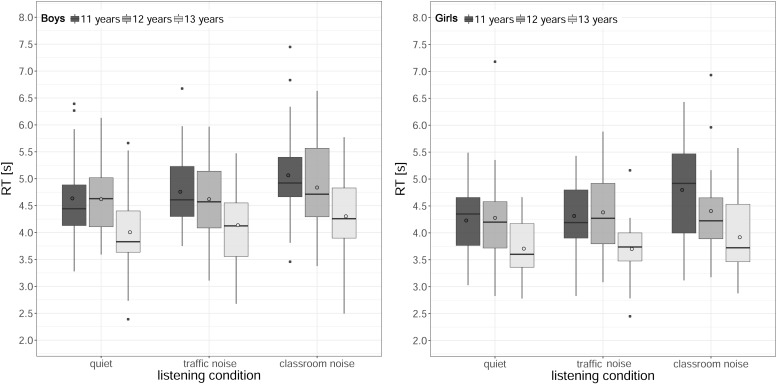
Boxplots of response times (RTs) in the sentence comprehension task by age and listening condition, for boys **(left)** and girls **(right)**. The length of the box corresponds to the interquartile range of the data distribution; the central, bold line is the median value, and the white circle is the mean; 99% of the data fall within the whiskers. Outliers are shown as black circles outside the whiskers.

Figure 7 shows the RTs in the SC task (median across the trials) for each listening condition and age, for boys and girls. The analysis identified a significant main effect of listening condition [χ^2^(2) = 30.64, *p* < 0.001], a main effect of age [χ^2^(2) = 25.68, *p* < 0.001], and a main effect of gender [χ^2^(1) = 7.21, *p* = 0.007]. The main effect of reading comprehension score was not significant (*p* = 0.051), nor were there any significant two- or three-way interactions (all *p*s > 0.38).

*Post hoc* tests comparing the listening conditions collapsed across age and gender showed that RTs were significantly slower in classroom noise than in quiet or traffic noise (quiet < classroom noise, *z* = −5.30, *p* < 0.001, ΔRT = 314 ms, traffic noise < classroom noise, *z* = −3.19, *p* < 0.001, ΔRT = 239 ms).

Comparisons between age groups, with data collapsed across listening condition and gender, revealed that RTs were faster for the oldest children (11 > 13 years, *z* = 4.95, *p* = < 0.001, ΔRT = 638 ms; 12 > 13 years, *z* = 3.24, *p* = 0.004, ΔRT = 543 ms). As for the effect of gender, the boys’ RTs were, on average, 319 ms longer than those of the girls.

### Comparison of the Effects of Background Noise and Age on the Two Tasks: RTs

**FIGURE 8:**
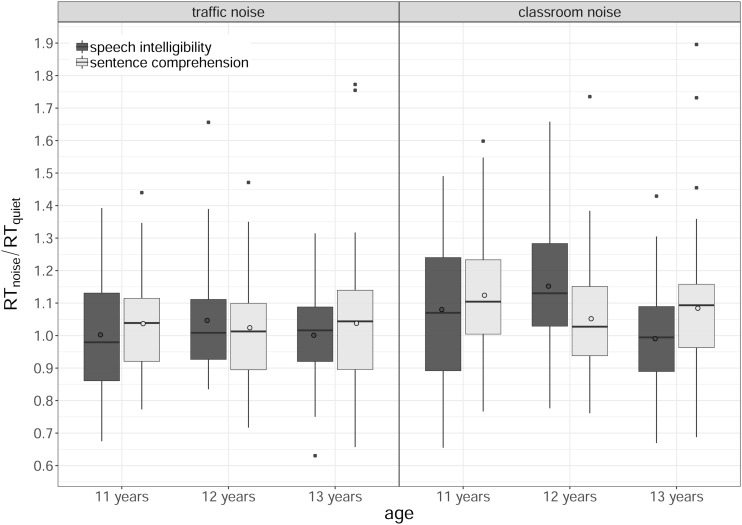
Boxplots of the normalized response times (RTs), by task (speech intelligibility, sentence comprehension), age (11, 12, 13 years) and listening condition (traffic noise, classroom noise). The length of the box corresponds to the interquartile range of the data distribution; the central, bold line is the median value, and the white circle is the mean; 99% of the data fall within the whiskers. Outliers are shown as black circles outside the whiskers.

Figure 8 shows the RT relative to quiet for each age group, task and noisy listening conditions (traffic noise, classroom noise). Our analysis found a significant main effect of listening condition [χ^2^(1) = 30.47, *p* < 0.001], a significant interaction between age and task [χ^2^(2) = 8.46, *p* = 0.015], a significant interaction between listening condition and age [χ^2^(2) = 8.09, *p* = 0.017], and a significant three-way interaction between listening condition, age and task [χ^2^(2) = 8.80, *p* = 0.012]. The main effects of age, gender, task, and baseline comprehension score, and the interaction between listening condition and task were not significant (all *p*s > 0.25).

As shown in Figure 8, the three-way interaction was due to a different impact of the two background noises, which depended both on the type of task and on the children’s age. For each age group and task, pairwise comparisons were run to analyze the effect of the listening condition. For the 11-year-olds, there was a significant difference between the two noisy listening conditions in both tasks, with traffic noise less invasive than classroom noise (speech intelligibility: *t* = −3.31, *p* = 0.006; sentence comprehension: *t* = −3.72, *p* = 0.001). For the 12-year-olds, the difference between the two listening conditions was only significant for SI (traffic < classroom noise, *t* = −4.31, *p* < 0.001), and no difference was found for the 13-year-olds (all *p*s > 0.25). Whenever a significant difference emerged, it always pointed to classroom noise having a greater impact (prompting a greater increase in RT) than traffic noise.

## Discussion

The main aim of this study was to compare SI and SC in lower middle-school students, under three listening conditions (quiet, traffic noise, and classroom noise). Children from 11 to 13 years old were tested to clarify the effects of background noise, whether and how they may be influenced by the listener’s age or gender, and whether SI and SC are affected differently. The main findings of our study are discussed below.

### Effects of Noise

For both the tasks administered, the children in our sample performed best, and had the fastest RTs in the quiet listening condition. Adding background noise at a sound pressure level typical of a working classroom generally reduced the students’ accuracy in the tasks and increased their listening effort (according to their slower RTs). When SI was considered, there was a main effect of listening condition on task accuracy that discriminated between the specific effects of each condition: classroom noise disrupted SI significantly more than traffic noise, which was still more impairing than quiet. In the SC task, on the other hand, a strong ceiling effect emerged for accuracy, probably attributable to the additional cues provided by the pictorial representation of the actions. The visual, closed-set format of the test allowed for the inclusion of sentences of different linguistic complexity, but strongly supported listeners trying to complete the task, making the SC task easier than the SI.

As expected, classroom noise impaired performance accuracy in the SI tasks more than traffic noise. The presence of speech-like temporal fluctuations in the masker adversely affects task performance accuracy in verbal tasks by competing with the target speech (Dockrell and Shield, 2006). It should be noted that even notionally steady-state maskers (like the traffic noise used in the present study) can produce modulation masking – which interferes with the target speech processing – for adult listeners (Stone et al., 2011, 2012), but there is no evidence of the same effect in children. The adverse effect of the classroom noise used in the present study may also relate to a capture of attention. In fact, salient sound events (like the events mixed with the ICRA signal) further impair performance accuracy by capturing the listener’s attention (Klatte et al., 2010b). This latter mechanism is known to depend on individual attentional abilities (Klatte et al., 2013), which may explain the greater variability in accuracy (i.e., larger standard deviations) seen in the SC task associated with classroom noise (see Table 4).

RTs were recorded to see whether the type of noise had the same effect on listening effort as on task performance accuracy. A main effect of listening condition on RTs was found in the SC task, indicating that the children took longer to process what they heard (240 ms) in classroom noise as opposed to quiet or traffic noise. A more complex pattern emerged for the SI task, for which a significant interaction emerged between listening condition and age. The RTs were slower in classroom noise than in quiet or traffic noise, but only for the 11- and 12-year-olds, not for the 13-year-olds. This would suggest a developmental effect on the strategies for coping with noise, which is discussed in more detail in the next section.

In the SC task, the children in our study were able to cope with traffic noise, which impaired neither their performance accuracy nor their RTs by comparison with the quiet condition. In the SI task traffic noise did not impair the children’s RT and only slightly decreased their performance accuracy (by 1.6 percentage points) by comparison with the quiet condition. In classroom noise, however, the increase in the 11- and 12-year-olds’ RTs reflected the worsening of the task performance accuracy. This finding is consistent with previous studies on children using RT as a behavioral proxy for listening effort (Prodi et al., 2013, 2019; Lewis et al., 2016; McGarrigle et al., 2019). The latency before a response includes the time listeners take to decode and process the auditory information they have received, so it can be considered informative on the effort invested in the task, or the cognitive resources needed to process the stimulus (Gatehouse and Gordon, 1990; Houben et al., 2013; McGarrigle et al., 2014; Pichora-Fuller et al., 2016). A slower RT is interpreted as a sign of a greater listening effort, and several studies have already found the measure sensitive to adverse conditions, such as a worsening of the SNR. More cognitive resources are needed to process auditory information in degraded listening conditions, leaving fewer resources available for the actual task, and leading to a weaker performance.

Overall, the findings of the present study support the existing literature on the harmful effects of background noise with a fluctuating temporal envelope and salient sound events on children performing SI and SC tasks (Klatte et al., 2010a; Prodi et al., 2013), confirming that this also applies to 11- to 13-year-olds.

### Effects of Age

Another question addressed in this study was whether children from 11 to 13 years old show any developmental effect on how they cope with background noise in SI and SC tasks. Our interest lay in investigating whether age interacted with type of noise and, if so, whether task performance accuracy and listening effort showed the same pattern of results.

Concerning SC, age had a significant main effect on RTs, the 13-year-old students always answering faster than the 11- or 12-year-olds: the former took 500 ms less time to process the sentences than the latter. This developmental effect in the SC task was unaffected by listening condition, as no interaction emerged between the two factors. This would suggest that the effect of age is due to more basic developmental processes, involving memory functioning or language competences, for instance. Sullivan et al. (2015) found that working memory and vocabulary size (both of which increase with age) contributed to children’s comprehension, in both quiet and noise.

It is also worth emphasizing that this difference in RTs in the SC task was seen despite a ceiling effect in the results for task accuracy. This result is in line with studies indicating that RTs may vary for the same level of task accuracy, and even when listeners have already reached their highest possible level of accuracy. Listening effort may therefore be a totally different construct from task performance accuracy. Several studies witnessed this effect for adults (Houben et al., 2013; Picou et al., 2013), but few have explored it in children (Sahlén et al., 2017; Prodi et al., 2019).

As for the SI task, performance accuracy was significantly lower for 11-year-olds than for the older children already in the quiet condition, and the same difference applied to the noisy conditions – as indicated by the absence of any interaction between age and listening condition. This finding might suggest that 11-year-olds found the ITAMatrix (administered in real classrooms using a fixed-stimuli procedure) more difficult than the older students. In the quiet condition, in which the extremely favorable SNR and the modest contribution of reverberation led us to expect the highest SI results, the 11-year-olds fared significantly worse than the older children, while the 12- and 13-year-olds reached a near-ceiling accuracy – possibly meaning that in a quiet condition an adult-like performance accuracy is acquired by 12 years of age. The age effect observed in the SI task would be in line with many published reports of the ability to perceptually segregate speech from a noise masker being immature in childhood, but adult-like by adolescence. For instance, Leibold and Buss (2013) found that adult-level performance accuracy was reached already at around 8 years old in a consonant identification task conducted in speech-shaped noise. A mature performance was observed a little later on, by about 9–10 years of age, in other studies (Corbin et al., 2016). This ability appears to develop at different rates, however, also depending on the characteristics of the masker (Wróblewski et al., 2012; Leibold, 2017), and on the stimulus type (Lewis et al., 2016).

When RTs in the SI task are considered, a picture complementary to task performance accuracy can be drawn. No effect of age was seen in quiet or in traffic noise, but in classroom noise the 13-year-olds’ RTs were significantly faster. Based on these results, the effects of age on SI in noise would depend on the nature of the masker for listening effort as well. The absence of an age effect in traffic noise could relate to the temporal characteristics of this masker, which is essentially steady-state, with no salient sound events that may capture a child’s attention (Klatte et al., 2013). Using a similar traffic noise and SI task, Prodi et al. (2013) found no difference in the RTs of children between 8 and 10 years old, but longer RTs for children aged 6 or 7. The similarity of the experimental setups enable the findings of the two studies to be compared. It may be that, by 8 years old, the presence of traffic noise during a SI task mainly impairs “bottom–up” processing, with less call for additional, explicit cognitive processing.

In classroom noise, there was a significant effect of age on RTs, with older students responding faster. Younger students are more susceptible to sound-induced distractors (e.g., salient sound events) due to their more limited attentional control (Klatte et al., 2010b, Klatte et al., 2013). This means that our 11- and 12-year-old children needed to dedicate more active resources to the task, and this increased their processing time. This finding confirms – and extends up to 12 years of age – a trend already seen in children 6 to 10 years old by Prodi et al. (2013): RTs were significantly slower, under the same masker, the younger the age of the respondent. No difference in RTs emerged between the two background noise conditions for our 13-year-old sample, suggesting that they had already developed the key cognitive abilities needed to cope with speech in noise. No adult group was included in our study, which could have served as a benchmark against which to compare the 13 year-olds’ results, and judge the age at which processing time may plateau. The age of 12 years seemed crucial to both accuracy and RTs in the SI task: this age group’s task performance accuracy was better than that of the younger children, and comparable with that of the older ones, but the 12-year-olds still needed more processing time than the 13-year-olds.

### Effects of Gender

Significant differences emerged in the present study between boys’ and girls’ task performance accuracy and RTs. In the SC task, girls always had shorter processing times than boys. The averaged RT gap was quite large (319 ms), representing 9.4% of the average duration of the COMPRENDO sentences. In the SI task, the girls were 2.2 percentage points more accurate than the boys, but their RTs were only significantly shorter (by 316 ms; 13.7% of the average duration of the ITAMatrix stimuli) at 13 years of age.

Our findings of a better performance in girls confirm the uneven developmental course of speech reception for males and females, and are in line with previous reports on accuracy (Ross et al., 2015). As gender no longer makes a significant difference when adult groups are considered (Ross et al., 2015), this effect may be driven by the development of underlying abilities in the age range considered here, and particularly by gender-related differences in the processing of verbal tasks (Burman et al., 2008; Etchell et al., 2018).

It is worth noting that despite the statistically significant main effect of gender on SI performance accuracy, the difference in the SI scores of male and female was very small (2.2 percentage points referred to a mean SI of 90.7%) and might have a limited relevance in the classroom setting. Differently, the present study shows that RTs can provide some interesting additional information, which have practical implications for the children’s performance in classrooms. An interaction between age and gender was found for the SI task, but was not significant for SC. When listening effort was considered, and the analysis was limited to the reception of multiple words (as in the SI tasks), the advantage of females was confined to the 13-year-old group. When a more comprehensive display of processing capacity was needed, however, as in the SC task, the gap between females and males applied at all the ages considered. Given the fast pace of communication in classrooms, and the amount of new information that pupils face during lessons, a slowing down in the processing time of the verbal message would likely have a negative impact on the students’ learning. In addition, the RT to a task give information on the effort invested, and an increase in RTs can be taken to reflect an increase in listening effort. A prolonged effort (as requested over the time of a lesson or over the school hours) may lead to an outcome of mental stress and fatigue, which is often associated with slower information processing, decreased level of goal-directed attention, difficulties in focusing on the task, and increased involuntary shifts of attention (Key et al., 2017).

It should be noted that the present RT results (referring to 11- to 13-year-old children) contrast with the report from Sahlén et al. (2017) of slower RTs for girls than for boys when 8-year-olds are considered. Given the similarity of the SC tasks employed in the two studies, the reasons for this discrepancy probably lie in the different age ranges considered, and the dysphonic voice used by Sahlén et al. (2017).

Finally, it is also worth noting that, both in the present study and in the one by Boman (2004), the effect of gender on task performance accuracy did not interact with the listening condition. This would suggest that the effect was not driven by a different sensitivity to noise, but by a more basic difference between the two genders in the 11–13 age range.

### Speech Intelligibility Versus Sentence Comprehension

This work compared SI and SC using a standardized audiological test for SI and a standardized test battery for SC. The two tests rely on different levels of speech processing. In the SC task, listeners first have to construct a coherent integrated mental representation of a sentence’s meaning by combining lexical, semantic and syntactic information; then they must choose the appropriate image on the screen after comparing with confusing competitors. In the SI task, listeners have to recognize and sequentially select all the words of a sentence, without contextual or semantic cues to support the recall phase. It would therefore be inappropriate to compare the absolute results of the two tasks directly, so changes in RT in noisy conditions relative to quiet were considered. Using normalized quantities, the additional negative effects of noise on response latencies in the two tasks were compared after the effects of age and gender had been partialled out of the analysis.

The results indicated that the type of noise affected RTs differently depending on the participants’ age. In particular, a significant three-way interaction was found between task, age and noise, reflecting a developmental effect on how the children coped with the more challenging classroom noise. This suggests that, when the burden on cognitive processes is considered, the comparison between the two tasks might be even more challenging than the one revealed by accuracy alone, as reported in previous studies. When SI and SC were compared in both adults (Hustad, 2008; Fontan et al., 2015) and primary school children (Klatte et al., 2007), SI scores proved to be poor predictors of comprehension performance accuracy in quiet conditions (Hustad, 2008). In addition, the two tasks were differently affected by background noise level (Fontan et al., 2015) and the spectro-temporal characteristics of the masker (Klatte et al., 2007). Generally speaking, transposing SI results (in quiet or in noise) directly to SC might not be meaningful, and acoustic conditions that guarantee optimal SI might not be equally adequate for SC. This issue needs clarification because most currently-used technical means for assessing room acoustics rely on SI, and have no clear and unambiguous connection with SC.

Judging from what we know for now, it does not seem that a simple relationship can capture the link between SI and SC tasks (as hypothesized, for instance, by Hygge, 2014), as it is strongly affected by the characteristics of the tasks themselves. The choice of using tasks based on different speech materials and the presence of a strong ceiling effect on the accuracy on SC task, prevented the possibility of directly exploring the relationship between SI and SC in the present study. However, the SC method applied here presents two main advantages: its easy pictorial implementation and the chance to obtain accuracy and RT data simultaneously – features that make the SC test appropriate for different categories of listeners, and students in particular.

### Study Limitations and Future Directions

The present study has some limitations. The hearing sensitivity was not measured for the children participating in the study, and the presence of possible hearing impairments was based only on the parent and teacher’s reports. In addition, the SC performance accuracy results showed a strong ceiling effect in all listening condition and for all ages. This happened despite the test being based on sentences of different lexical difficulty. Given the limited number of sentences in each list, a reliable statistical analysis including complexity as an explanatory variable could not be pursued. That said, exploratory analysis suggested a significant trend of declining performance accuracy (and slowing RTs) with increasing sentence difficulty. Aiming to investigate the effect of syntactic complexity and its possible interaction with the noise type, future studies might consider more sentences for each complexity level and include the sentence difficulty as a factor in the analysis of the task performance accuracy.

The near-ceiling results also prevented any direct comparison between SC and SI, as concerns performance accuracy. The interactions identified by our analysis on the normalized RTs give us the impression that a more extensive comparison would be worthwhile. In particular, it would be important to explore a wider range of reverberations and SNRs, using maskers comprising more competing talkers or intelligible speech. These manipulations would improve our understanding of the objective characteristic of maskers that mediate the relationship between the two tasks.

The results of our study indicate that the ITAMatrix may not be suitable for 11-year-old children in classrooms, because they were unable to perform as well as the 12- and 13-year-olds even in quiet condition. The reasons behind this finding warrant further investigation, the first step being to see whether the same pattern of results is seen at this age in anechoic conditions too. It may be that this age group would manage better with the simplified version of the Matrix Sentence Test (with three- instead of five-word sentences). The applicability of the simplified ITAMatrix has been demonstrated in clinical settings for children 5 to 10 years old (Puglisi et al., 2018), and in both noisy and anechoic conditions the performance of 10-year-olds already approached that of adults. Using this simplified test for older pupils (12–13 years old) as well would level the task difficulty between the age groups. Finally, Puglisi et al. (2015) established the presence of a practice effect when the ITAMatrix is presented in a clinical setting, using an adaptive procedure converging at a SI = 50%; two test lists of 20 sentences are recommended to account for the effect. In the present study higher SI values were targeted (due to the realistic listening conditions selected for the experiment), a constant stimuli paradigm was used, and the test was presented collectively and not at the individual level. Given the much simpler procedure than in a clinical setting, the children were expected to accustom to it more easily reducing the practice effect, and only four sentences were presented during the training phase of the task. Even though the potential presence of training effects was addressed by counterbalancing the listening conditions among the classes, there might be remaining training effects depending on the age of the children.

## Conclusion

The present study provides evidence that supports previous reports, and also better frames the relationships between type of noise, age, gender, and task. The main results can be summarized as follows.

Effects of age and listening condition were found mainly for the SI task, on both accuracy and RTs. The most demanding condition was in classroom noise, when the SI scores were lowest and the RTs slowest. In this condition, 11- and 12-year-olds needed the same processing time, but the former group scored lower for accuracy. The 12-year-olds already performed as well as the 13-year olds in terms of accuracy, but with slower RTs. The oldest students had the fastest RTs. A pattern for SI thus emerged, with improvements in task performance accuracy preceding improvements in processing time. This is consistent with findings in younger children and presumably due to a mechanism whereby the cognitive processes underpinning speech reception are first acquired and later consolidated. In the SC task, accuracy scores neared the ceiling, meaning that merging accuracy and RT data was not as informative as in the SI task.

This study also confirmed the effects of gender on the SI and SC tasks. In particular, a main effect of gender was found on the latter task, indicating that the gap between girls and boys was wider for the task of greater linguistic complexity that engaged the pupils in a listening situation more closely resembling actual communication in classrooms. Standardized tests should be developed to include the assessment of this competence when designing for classroom acoustics. Mitigating the gender bias in SC could prove difficult, however, as it may involve class management and how classes are organized.

Finally, our study showed that classroom noise slowed response latencies by comparison with the quiet condition in both SC and SI. Since several factors – such as the nature of background noise, and children’s age – appear to affect differently the two tasks, it will be necessary to develop specific test settings to investigate a possible model linking SC and SI.

## Data Availability Statement

The datasets generated for this study are available on request to the corresponding author.

## Ethics Statement

This study was approved by the Ethics Committee of the University of Padova (Italy). Written informed parental consent was obtained prior to the test.

## Author Contributions

NP and CV conceived the study, designed the experiment, and managed contacts with the schools, took care of the data collection, and wrote the first draft of the manuscript. EB and IM advised on the experimental design, developed the children’s baseline assessment, and calculated the related statistics. AD provided the sentence comprehension tests used in the study. CV performed the statistical analysis. All the authors participated in refining the data analysis by means of group discussions, added sections of the manuscript, and revised the whole text up until final approval.

## Conflict of Interest

The authors declare that the research was conducted in the absence of any commercial or financial relationships that could be construed as a potential conflict of interest.
